# The Transtheoretical model for fruit, vegetable and fish consumption: associations between intakes, stages of change and stage transition determinants

**DOI:** 10.1186/1479-5868-3-13

**Published:** 2006-06-19

**Authors:** Emely De Vet, Jascha de Nooijer, Nanne K de Vries, Johannes Brug

**Affiliations:** 1 Department of Health Education and Health Promotion, Universiteit Maastricht, PO Box 616, 6200 MD Maastricht, The Netherlands; 2 Department of Public Health, Erasmus MC University Medical Center, PO Box 2040, 3000 CA Rotterdam, The Netherlands

## Abstract

**Background:**

Cardiovascular diseases are caused by multiple behavioral factors, including different dietary factors. We examined to what extent fruit, vegetable and fish consumption are related, and whether behavioral determinants vary across these dietary behaviors from a Transtheoretical model perspective.

**Methods:**

Data were collected among 1142 participants (T0; response rate 46%) selected from an Internet panel, who were followed-up one-week later (T1; N = 1055, response rate 92%). Mean age was 35.4 (SD = 11.9) years, 35% was male, and most respondents were of Dutch origin (90%). Of the respondents, 13%, 44% and 43% had a low, medium or high level of education, respectively. Electronic questionnaires assessed fruit, vegetable and fish intake (food frequency questionnaires), stages of change, decisional balance and self-efficacy, for each of these three behaviors.

**Results:**

Stages of change and (changes in) fruit, vegetable and fish intake were only weakly associated; decisional balance and self-efficacy were more strongly associated. Some presumed predictors of stage transitions were similar for fruit, vegetable, and fish intake, i.e., strong pros predicted progress out of precontemplators and low self-efficacy predicted relapse from action/maintenance for all behaviors. However, progress out of contemplation and out of preparation showed different patterns for fruit, vegetable and fish intake.

**Conclusion:**

The weak associations between intakes and potential determinants for fruit, vegetable, and fish consumption do not warrant an integrated dietary change approach targeting the same determinants for each behavior.

## Background

Cardiovascular diseases (CVD) are major causes of morbidity and mortality in most Western countries [1], including the Netherlands [2]. CVD risk is influenced by multiple behaviors among which different dietary behaviors, e.g., fruit, vegetable and fish consumption [1]. To identify high-risk groups and to understand and change multiple behaviors, research focusing on multiple dietary behaviors at the time is frequently advocated [3,4]. To date, most health behavior change research tends to study or target one behavior in isolation [5,6]. Further, studies that are concerned with multiple behaviors are mainly concerned with the co-occurrence of behaviors, and do not study the co-occurrence of behavioral determinants of behavior [7]. Therefore, we examined to what extent fruit, vegetable, and fish consumption and their determinants are associated, and whether fruit, vegetable and fish consumption are influenced by the same behavioral determinants.

In many countries, fruits and vegetables are considered one food category, and hold one recommendation for both fruits and vegetables (e.g., the 5 a day recommendation in the USA). However, fruits and vegetables are commonly eaten at different occasions and it has been found that behavioral determinants differ for fruit and vegetables intake [8]. Therefore, in the Netherlands, separate recommendations are provided for fruit (two servings of fruit each day, which corresponds to 250 grams a day) and vegetables (200 grams each day). The amount of adequate intake for n-3 fatty acids is set at 0.2 grams per day [9]. To achieve this intake, fish is recommended once or twice a week in the Netherlands (approximately 175 grams a week). All sorts of fish, i.e. fresh, processed and tinned fish are included. Since crustaceans (e.g. shrimps) and shellfish (e.g. mussels) are also a source of n-3 fatty acids, these products may be eaten to achieve the recommended intake as well [9,10]. Consumption levels of fruits, vegetables and fish are below recommended intake levels in many countries, including in the Netherlands. To develop effective interventions to increase intake levels, determinants of fruit, vegetables and fish consumption should be identified [11].

One of the most popular models for studying behavioral determinants and informing interventions is the Transtheoretical model of behavior change (TTM) [12,13]. The TTM defines five discrete stages of change (i.e., precontemplation, contemplation, preparation, action and maintenance), which discriminate people based on current behavior and the intention to change this behavior. In the precontemplation stage, people are not motivated to change their unhealthy behavior within the next six months, while maintenance reflects the stage in which people have been behaving healthy for longer than six months. According to the TTM, people can progress and regress from one stage to another. Such movements are called stage transitions. Specific TTM components, such as decisional balance or self-efficacy, should be applied in interventions in order to facilitate movement to further stages of change. Decisional balance refers to an individual's relative weighing of pros and cons [14,15]. The TTM hypothesizes that, to progress from precontemplation, the pros of changing must increase. For progress from contemplation, the cons of changing must decrease. Self-efficacy reflects the situation-specific confidence people have that they can cope with high-risk situations without relapsing. The TTM assumes that self-efficacy increases monotonically from precontemplation to maintenance [16].

The TTM may be a useful framework for research on multiple behaviors, since the TTM assumes that the process of behavior change is not problem-specific, i.e., the principles of the TTM can be applied to any behavior [15,17]. As a consequence, TTM-based interventions would target pros, cons and self-efficacy to produce stage progress, independent of the target behavior [17]. However, since longitudinal research on determinants of stage transitions in dietary behaviors is still largely lacking [18,19], it remains unclear whether pros, cons and self-efficacy are of equal importance in stage transitions for fruit, vegetable and fish consumption. Combined with the lack of studies focusing on various dietary behaviors and their determinants, the rationale for the present study was two-fold:

1. Do fruit, vegetable and fish intake, and their stages of change cluster?

If so, tailoring interventions for multiple behaviors would be better possible, since high-risk groups can be identified.

2. Are the presumed stage transition predictors, i.e. decisional balance and self-efficacy, of similar importance across stages of change for fruit, vegetable and fish intake?

If so, it is supportive for the TTM as a basis for developing interventions to change multiple behaviors.

## Methods

### Participants and procedures

Potential participants were recruited via a Dutch Internet panel sized 15,000 members at the time of study. Individuals had become a panel member in the past on the website of Flycatcher Internet research . By becoming a panel member they indicated their willingness to participate in various types of online research (e.g. online surveys or opinion polls) on all kinds of different topics (e.g. politics, marketing research, health issues). Respondents are not precontacted, but questionnaires are usually sent immediately to a selected sample. In the present study, a total of 2500 adults, randomly selected from the total panel, were invited to participate. They were sent an email letter, explaining the study details, with a link to the first electronic questionnaire. Respondents could indicate their willingness to participate in the study by completing the first electronic questionnaire (T0; N = 1142, response rate 46%). Exactly one week after completing T0, respondents received a second questionnaire (T1; N = 1055, response rate 92%). Mean age was 35.4 (SD = 11.9) years and 35% was male. Respondents who had completed no formal education, primary school, secondary school, and lowest level of high school or lower vocational training were classified as having a low level of education (13%). Respondents with a medium level of education had completed intermediate or high level high school, or medium level vocational training (44%). Respondents who had completed higher vocational training, college or university training had a high level of education (43%). Most respondents were ethnic Dutch (90%). As indicated by t-tests and chi-square tests, panel members who did not respond to the first questionnaire were significantly younger (*t *= 5.98, *p *< .001; non-responders' mean age = 32.6, SD = 11.2) and more often male (χ^2 ^= 16.83, *p *< .001; 43% of non-responders were male) than responders. Of the responders, no significant differences in age, sex, ethnicity, level of education, consumption levels or stages of change for fruit, vegetable or fish consumption were found between those who did complete both questionnaires and those who did not.

### Measures

#### Dietary behaviors

The three dietary behaviors were measured using food frequency questionnaires (FFQ) asking "how often did you consume the listed products in the past week?" (ranging from [not consumed] to [seven days]) and "on a day you consumed the listed product, how much did you take on average on that day?" (in pieces, bowls, or serving spoons). For *Fruit consumption *seven separate (groups of) products were listed reflecting the most common fruits in the Netherlands, i.e., citrus fruit (oranges, lemons, grapefruit or other citrus fruit), apples and pears, bananas, freshly squeezed or unsweetened fruit juice, tangerines, applesauce, and other fruits (including preserved fruit). *Vegetable consumption *was assessed with two separate questions for raw (e.g., lettuce, cucumber, tomato) and prepared vegetables (e.g. cooked, steamed, stir-fried, including preserved vegetables). For *fish consumption *six separate (groups of) products were listed for the most common fish, fish products and other seafood in the Netherlands, i.e. ready-made fish (e.g., fish sticks, fried haddock filet, cod parings), crustacean and shellfish (e.g., shrimps, crab, mussels), tinned fish (e.g., tuna, salmon, sardines), steamed, grilled, cooked or baked fish with main course (e.g., cod, pollack, plaice, sole, perch, including fresh fish as well frozen fish), mackerel or eel, and herring.

The FFQs used to assess fruit and vegetable consumption have been validated as compared to 7-day dietary records and biomarkers for fruit and vegetable consumption levels, and the FFQ for fish consumption has been validated against 3-day 24-hour dietary records [see for a detailed description of validity estimates; [20-22]].

Total weekly consumption in grams was calculated by multiplying the amount of consumption with standard mean weights, which were obtained from the Dutch food composition table [23]. Since fruit and vegetables are recommended on a daily basis, daily consumption in grams was calculated by further dividing weekly consumption by seven.

#### Stages of change

In line with recommendations from a study on staging instruments [24], and consistent with studies reported by Armitage and Arden [25] and De Vet, De Nooijer, De Vries, and Brug [26], stages of change for fruit, vegetable and fish consumption were assessed with one-item staging instruments with a five-choice response format. First, a description of the Dutch recommendations for each of the dietary behaviors was presented to ensure a correct understanding of the target behavior. Next, respondents were asked whether they met the recommended intake levels for the dietary behaviors, by selecting one of five statements each representing a stage of change: "No, and I do not intend to change this within the next six months" [precontemplation], "No, but I intend to change this within the next six months" [contemplation], "No, but I intend to change this within the next month" [preparation], "Yes, and I have started doing so in the last six months" [action], "Yes, and I have done so for more than six months" [maintenance].

#### Decisional balance

Decisional balance was assessed by asking respondents how important each of the listed pros and cons was in their decision to eat recommended amounts of fruit, vegetables or fish using five-point Likert scales ranging from -2 (*not at all important*) to 2 (*very important*). Decisional balance was assessed with eight pros and nine cons for fruit intake, and with eight pros and ten cons for vegetable intake. The decisional balance measures for fruits and vegetables were based on Ma et al. [27]. For fish intake, decisional balance was assessed with seven pros and eleven cons, based on instruments from others [28-30]. The Cronbach's α for pros and cons were satisfying and mean scores for pros and cons were computed (See Appendix for questionnaire items and Cronbach's α's).

#### Self-efficacy

Self-efficacy was assessed by asking respondents to rate on five-point Likert scales ranging from -2 (*very difficult*) to 2 (*very easy*), how difficult or easy they find it to eat according to the recommendations in six high-risk situations, for each of the three dietary behaviors [cf. [31]] (see Appendix).

### Data analyses

First, one-way analysis of variance (ANOVA) with Tukey HSD post-hoc tests were conducted to test differences in fruit, vegetable, and fish consumption between the baseline stages of change for fruit, vegetable, and fish, respectively.

Second, to examine the relation between dietary behaviors (study's first aim), Pearson *r *correlations were calculated for 1) fruit, vegetable and fish consumption levels at T0, 2) changes in consumption of fruit, vegetable, and fish between T0 and T1, and 3) decisional balance and self-efficacy for the three behaviors. To examine the relation between stages of change for the three dietary behaviors, Spearman's *ρ *correlations were calculated. According to Cohen's guidelines for interpretation of correlations, a large effect size was defined as a correlation larger than or equal to .50. A correlation between .30 and .50 is regarded as a medium effect size, and a correlation between .10 and .30 is defined as a small effect size [32]. T-tests were used to test differences in consumption levels between respondents who were in the same stage of change for all three behaviors (e.g. in precontemplation for fish, fruit and vegetable intake) and respondents who were in different stages for all three behaviors (e.g. in precontemplation for fish, in contemplation for fruit, and in action for vegetable intake).

Third, the role of TTM variables (pros, cons, and self-efficacy) in stage transitions was examined (study's second aim). For this purpose, for precontemplation, contemplation, and preparation at baseline a dichotomous forward stage transition variable was created (1 = at least one stage progress between baseline and follow-up; 0 = no progress). A dichotomous backward stage transition variable was created for respondents in action/maintenance at baseline (1 = relapse to precontemplation, contemplation, or preparation, 0 = no relapse). Logistic regression analyses were conducted with forward stage transition out of precontemplation, contemplation, preparation, and backward stage transition out of action/maintenance between T0 and T1 as dependent variable. Pros, cons, and self-efficacy at T0 were separately entered as independent continuous variables. The analyses were conducted for determinants and stage transitions related to fruit, vegetable and fish consumption, separately (e.g., pros of fruit intake are only included in the analyses to predict stage transition for fruit intake). Odds ratios (OR) were computed as the effect size estimate. Although no clear interpretation guidelines for the magnitude of OR exist, an OR near two is usually interpreted as meaningful [33].

All analyses were conducted using SPSS 11.0. Alpha levels of .05 were used for all statistical tests.

## Results

### Baseline consumption levels and stages of change for fruit, vegetable and fish intake

At baseline, daily fruit and vegetable and weekly fish consumption averaged 259 (SD = 163), 128 (SD = 83) and 148 (SD = 180) grams. For fruit and vegetable intake, the most frequently reported stage of change was maintenance (34% and 45%, respectively), while precontemplation was the most frequently reported stage of change for fish intake at baseline (40%, Table 1).

**Table 1:** Stages of Change and Average Fruit, Vegetable, and Fish Intake at baseline

	**Fruit**^†^		**Vegetable**		**Fish**	
	**N (%)**	**M (SD)**	**N (%)**	**M (SD)**	**N (%)**	**M (SD)**
PC	285 (25.0%)	167 (125)	267 (23.4%)	97 (62)	460 (40.3%)	55 (107)
C	202 (17.7%)	190 (135)	199 (17.4%)	90 (60)	146 (12.8%)	114 (96)
PR	144 (12.6%)	235 (146)	113 (9.9%)	101 (52)	71 (6.2%)	133 (172)
A	98 (8.6%)	319 (176)	49 (4.3%)	153 (116)	90 (7.9%)	222 (170)
M	413 (36.2%)	345 (146)	514 (45.0%)	162 (88)	375 (32.8%)	265 (191)

Model *F*		88.83***		53.13***		98.74***
Tukey HSD	PC, C < PR < A, M		PC, C, PR < A, M		PC < C, PR < A, M	

Note. *** *p *< .001

PC = precontemplation, C = contemplation, PR = preparation, A = action, M = maintenance

Total sample size N = 1142

M = Mean; SD = Standard Deviation

^† ^Fruit and vegetable intake in grams per day, fish intake in grams per week.

ANOVA showed significant differences in fruit, vegetable and fish consumption across stages of change. Tukey HSD post-hoc tests revealed that respondents in all pre-action stages consumed significantly less fruits, vegetables and fish than respondents in action and maintenance. Furthermore, with respect to fruit consumption, precontemplators and contemplators had a significantly lower intake level than preparators, whereas for fish consumption precontemplators consumed significantly less fish than contemplators and preparators. No significant differences were found between respondents in pre-action stages for vegetable consumption (Table 1).

### Associations in intake and stages of change between fruit, vegetable and fish

At T0, the dietary behaviors were weakly, but significantly correlated (*r *= .23 for fruit and vegetable, *r *= .16 for fruit and fish, and *r *= .16 for vegetables and fish; all *p *< .001). Changes in consumption between T0 and T1 were also weakly and often not significantly associated, i.e., *r *= .11 (*p *= .001) for changes in fruit and vegetable consumption, *r *= .06 (*p *= .06) for changes in fruit and fish consumption, and *r *= .04 (*p *= .20) for changes in vegetable and fish consumption.

Stages of change for the three dietary behaviors were weakly associated at T0 (Spearman's *ρ *= .20 for stages of fruit and vegetable, Spearman's *ρ *= .13 for stages of fruit and fish, and Spearman's *ρ *= .13 for stages of vegetable and fish; all *p *< .001). In total, 26% (n = 291) of the respondents were in different stages for fruit, vegetable and fish consumption, while 18% (n = 203) of the respondents were in the same stage for fruit, vegetable, and fish consumption. Respondents who were in the same stage for all three behaviors were most frequent in maintenance (55%, n = 111). T-tests further showed that respondents who were in the same stage for all three behaviors ate more fruits (*t *= -2.59, *p *= .01, Cohen's *d *= 0.25), vegetables (*t *= -3.47, *p = *.001, Cohen's *d *= 0.34) and fish (*t *= -4.79, *p *< .001, Cohen's *d *= 0.48) than respondents who were in different stages for these behaviors.

Pros of fruits and of vegetables, and the cons of fruits, of vegetables and of fish were strongly positively associated (*r *> .50; see Table 2). Moderately positive correlations (.30 <*r *< .50) were found between pros of fruits and of vegetables with the pros of fish, between the pros of fish and self-efficacy for fish, and between the self-efficacy for fruits and for vegetables. Further, the cons of vegetables and self-efficacy for vegetables were moderately negatively associated (Table 2).

**Table 2:** Pearson *r *Correlations between Pros, Cons, and Self-efficacy for Fruit, Vegetable and Fish consumption

	1	2	3	4	5	6	7	8	9
Pros vegetable (1)	1	.13***	.82***	.15***	.40***	.18***	.21***	.20***	.04
Cons vegetable (2)		1	.16***	.74***	.05	.53***	-.31***	-.12***	-.14***
Pros fruit (3)			1	.15***	.39***	.19***	.13***	.26***	.02
Cons fruit (4)				1	.08*	.55***	-.17***	-.24***	-.05
Pros fish (5)					1	.32***	.09**	.09**	.49***
Cons fish (6)						1	-.13***	-.12***	-.17***
Self-efficacy vegetable (7)							1	.45***	.25***
Self-efficacy fruit (8)								1	.22***
Self-efficacy fish (9)									1

Note. * *p *< .05, ** *p *< .01, *** *p *< .001

### Predictors of stage transitions between T0 and T1

The prevalence of stage transitions, i.e., regress, stability, or progress, is depicted in Table 3.

**Table 3:** Prevalence of stage transitions between baseline and follow-up for fruit, vegetable and fish consumption

	**Stage of change at baseline**			
**Stage transition**	**PC**^**a**^	**C**	**PR**	**A/M**

**Fruit**				
Regress	---^b^	*29 (16%)*^*c*^	*41 (32%)*	81 (17%)
Stable	189 (71%)	97 (54%)	52 (41%)	400 (83%)
Progress	77 (29%)	54 (30%)	53 (27%)	---
**Vegetable**				
Regress	---	*33 (18%)*	*39 (38%)*	90 (17%)
Stable	177 (73%)	111 (60%)	33 (33%)	434 (83%)
Progress	67 (27%)	42 (22%)	29 (29%)	---
**Fish**				
Regress	---	*36 (26%)*	*37 (54%)*	77 (18%)
Stable	357 (84%)	69 (51%)	16 (23%)	350 (82%)
Progress	67 (16%)	31 (23%)	15 (22%)	----

Note. ^a ^PC = Precontemplation, C = Contemplation, PR = Preparation, A/M = Action/maintenance

^b ^Note that it is impossible to regress from precontemplation or to progress from action/maintenance

^c ^Numbers printed in italics are not included in the analyses of predictors of stage transitions

#### Predictors of forward stage transition out of precontemplation

Precontemplators who rated the pros of fruit, vegetable and fish consumption as more important, were more likely to progress to a further stage for fruit, vegetable, and fish consumption, respectively. Further, higher self-efficacy significantly predicted forward stage transition out of precontemplation for fruit and fish intake, and marginally for vegetable intake. Cons did not predict forward stage transition out of precontemplation for the three dietary behaviors (Table 4).

**Table 4:** Predictors of Stage Transitions between T0 and T1 for Fruit, Vegetable and Fish intake

	**Fruit**		**Vegetable**		**Fish**	
	**OR**	**95% CI**	**OR**	**95% CI**	**OR**	**95% CI**
*Predictors of Forward Stage Transition out of Precontemplation*						
Pros	2.53***	1.59–4.00	2.42***	1.43–4.07	2.76***	1.89–4.04
Cons	1.01	0.69–1.48	1.01	0.65–1.56	0.90	0.67–1.22
Self-efficacy	1.69**	1.22–2.35	1.51^†^	0.98–2.34	1.84***	1.33–2.54
*Predictors of Forward Stage Transition out of Contemplation*						
Pros	1.32	0.75–2.31	1.38	0.66–2.89	1.53	0.63–3.73
Cons	1.04	0.63–1.71	0.57*	0.33–1.00	0.93	0.50–1.73
Self-efficacy	1.53^†^	0.93–2.52	2.26*	1.19–4.29	2.37*	1.0–95.16
*Predictors of Forward Stage Transition out of Preparation*						
Pros	0.91	0.40–2.05	0.91	0.33–2.50	0.58	0.19–1.81
Cons	0.87	0.44–1.75	0.69	0.28–1.71	0.57	0.20–1.62
Self-efficacy	2.71**	1.34–5.48	4.13**	1.53–11.15	2.28	0.67–7.69
*Predictors of Backward Stage Transition out of Action/Maintenance*						
Pros	0.71	0.45–1.11	0.93	0.62–1.40	0.83	0.54–1.29
Cons	1.27	0.90–1.80	1.70**	1.19–2.42	1.57*	1.07–2.31
Self-efficacy	0.39***	0.28–0.56	0.38***	0.26–0.56	0.34***	0.23–0.51

Note. ^† ^*p *< .10, * *p *< .05, ** *p *< .01, *** *p *< .001

OR= Odds ratio; 95% CI = 95% Confidence Interval

#### Predictors of forward stage transition out of contemplation

For all three behaviors, pros did not predict forward stage transition out of contemplation. Contemplators, who rated the importance of cons of vegetable intake as less important, were more likely to progress out of contemplation for vegetable intake. Cons did not predict progress out of contemplation for fruit and fish intake. Contemplators with high self-efficacy were more likely to move forward through the stages of change, for vegetable and fish, but only marginally for fruit consumption (Table 4).

#### Predictors of forward stage transition out of preparation

For fruit, vegetable as well as fish consumption, pros and cons did not predict progress from preparation. For fruit and vegetable consumption, but not for fish consumption, preparators with high self-efficacy were more likely to progress (Table 4).

#### Predictors of backward stage transition from action/maintenance

For none of the dietary behaviors, pros predicted relapse from the action/maintenance stages. For vegetable and fish consumption, but not for fruit consumption, relapse from action/maintenance was more likely if cons were rated as important. Further, low self-efficacy scores predicted relapse from action/maintenance for fruit, vegetable as well as fish consumption (Table 4).

## Discussion

Although various health behaviors may contribute to the prevention of CVD, health behavior research mainly focuses on understanding or changing single behaviors. We attempted to gain insight into the associations between three CVD preventive dietary behaviors, i.e. fruit, vegetable and fish intake. The results showed that 1) (stages of change for) fish, fruit, and vegetable intake, but particularly the behavioral determinants (decisional balance and self-efficacy) were related, and 2) pros predicted progress out of precontemplation and self-efficacy predicted relapse out of action/maintenance for all three behaviors, but the pattern of predictors as hypothesized by TTM was confirmed only for vegetable intake.

With respect to the first main finding, the correlations between the dietary behaviors were not very strong. However, taking into account the validity of the frequency questionnaires and the small amount of change within only one week, the correlations may not be considered weak. Also, the correlations were generally higher than those found in previous research on clustering of health behaviors. For example, Kremers and coworkers [7] showed that fruit consumption, less snacking, less high-fat sandwich fillings, active transport and physical activity correlated from -.01 to .14. In their view, behaviors and behavioral determinants might be more closely related within a behavioral domain (i.e. between dietary practices) than between behavioral domains (i.e. between diet and exercise)[7]. The three nutrition behaviors are expected to relate, since they constitute parts of a larger dietary pattern, the food products may be consumed in combination, and all behaviors may be associated with health benefits. Strongest relations were found between the (determinants of) consumption of fruits and vegetables. Three explanations may be given. First, fruits and vegetables are both recommended on a daily basis, whereas fish is recommended on a weekly basis. Second, fruits and vegetables are often promoted as a single food group. Third, fish has gained much less attention in the media or from health promoting agencies than fruits and vegetables. As a consequence, people may be less familiar with the recommendations for fish, than for fruits and vegetables. Some of the attention to fish even stressed presumed contaminant-related health threats from fish instead of the health promoting effects, which in turn may have affected people's beliefs about fish. Such contradicting information may result in attitudinal ambivalence, which may hinder progress to more advanced stages of change [25,34]. Indeed, we found that pros and cons for fish were positively correlated, indicating that people may simultaneously hold positive and negative beliefs towards fish resulting in ambivalent attitudes.

With respect to the second main finding, our results do not fully support the TTM, since pros, cons, and self-efficacy did not consistently predict similar transitions across different behaviors. Remarkably, only for vegetable intake the pattern of predictors as hypothesized by TTM was confirmed. Cons, for example, did not predict any of the stage transitions for fruit intake, consistent with earlier longitudinal research [31]. However, cons predicted forward transition out of contemplation for vegetable intake and relapse from action for vegetable and fish intake. It might be that these behaviors have more presumed disadvantages than the consumption of fruits. The fact that the cons are of particular importance for relapse may indicate that once people try to change their diets, they may encounter negative experiences with maintaining their new dietary habits. So, interventions aimed at changing vegetable and fish consumption may aim to reduce the importance of negative beliefs that arise *after *behavior change.

Some limitations of our study must be considered. First, a time interval of one week may be considered too small to study stage transitions and behavioral changes, especially with respect to fish consumption as fish is recommended on a weekly basis. However, according to the TTM, stages are considered states and individuals can move rapidly between stages, even within a single session intervention [p. 1046, [35]]. A previous longitudinal study for fruit intake showed that stage transitions also occur on a short notice, e.g. within three days [26]. In that study, it is argued that short-term stage change may be explained by 1) real self-change, 2) unreliable stage measurement, or 3) the idea that psychological constructs may vary over time [26]. Our results showed that stage transitions were predicted by decisional balance and self-efficacy, indicating that unreliable stage measurement is unlikely. Second, the instruments for decisional balance and self-efficacy were more similar for fruits and vegetables than for fish. This may have induced the stronger relation between fruits and vegetables than between these behaviors and fish. Fish is much less frequently studied, and only few tested questionnaires were available, and further research on determinants of fish consumption is necessary. Third, our sample may not be fully representative for the general Dutch adult (Internet) population, which may be due to the data-collection method, i.e. using an Internet panel. The response to the initial invitation was not optimal (46%), although our response was in line with results from meta-analyses on response rates, showing an average response rate of 40% for web- and Internet based questionnaires [36] and 49% for postal questionnaires [37]. Compared to the Dutch population at large, in our sample respondents with a higher education level, of Dutch origin, under 40 years of age, and females were over-represented [38]. The low participation rate among males may also be subscribed to the study topic. Research suggested that men are less likely to have responsibility for food purchasing and preparation and they may therefore have a weaker interest in messages and surveys on diet [39]. An advantage of the Internet panel was that we were able to include precontemplators and contemplators, who are normally difficult to reach. In our study, 55%, 51% and 60% of the respondents were in one of the pre-action stages of change for fruit, vegetable and fish consumption, respectively. Previous studies on stages of change and dietary behaviors showed lower numbers in these stages [40-42], e.g. only 27% of participants were in these early stages for fruit and vegetable consumption [42].

What do our results suggest for practice and future research? Our results do not warrant an integrated dietary change approach. The TTM defines targets for intervention, which should be applicable for virtually any behavior. According to our results, it may not be possible to generalize across behaviors and to apply the same TTM intervention components for various behaviors. It may even complicate the effectiveness of an intervention, since people may receive redundant information (e.g. information about the cons of fruit intake). Two randomized controlled trials have tested TTM-based multiple-behavior interventions [5,43]. Prochaska and colleagues [43] showed that targeting physical activity and nutrition simultaneously was equally effective to targeting physical activity only [43], but it has been suggested that changing multiple risk behaviors simultaneously may require too much effort of an individual [44]. Vandelanotte and colleagues [5], however, reported no substantial differences in effects between an intervention targeting physical activity and nutrition either simultaneously or sequentially. Future research should point out by which mechanism, people change multiple behaviors and how interventions can be adapted to this mechanism.

## Conclusion

We conclude that although TTM components, stages of change and fruit, vegetable and fish consumption seem to be somewhat related, predictors of stage transitions appear to have different impacts for different dietary behaviors. Therefore, we recommended that before developing a (multiple behavior) stage-based intervention, relevant determinants of each target behavior should be examined.

## Competing interests

The author(s) declare that they do not have any competing interests.

## Authors' contributions

EV directed the aspects of the study, including development, assessment and analyses, and led the writing of the manuscript. JN helped developing measures, designing the study and assisted writing the manuscript. NKV and JB both assisted with the writing of the manuscript. All authors read and approved the final manuscript.

**Appendix:** Decisional balance and self-efficacy questionnaire items

**Concept**	Items	**Cronbach's α**
**Below several pros and cons of eating (fruits/fish/vegetables) are listed. How important are each of these pros and cons in your decision to eat two servings of fruit each day/200 grams of vegetables each day/fish once or twice a week**		
Pros of fruit	Fruit is good for your body	.79
	Other people eat fruit	
	Fruit can substitute for unhealthy food products	
	Eating fruits can help one maintain weight	
	Fruit can help prevent diseases	
	Eating fruits makes one feel better	
	Fruits make a diet more varied	
	Eating fruits can help one lose weight	
Pros of vegetable	Vegetables are good for your body	.75
	Other people eat vegetables	
	Vegetables can substitute for unhealthy food products	
	Eating vegetables can help one maintain weight	
	Vegetables can help prevent diseases	
	Eating vegetables makes one feel better	
	Vegetables make a diet more varied	
	Eating vegetables can help one lose weight	
Pros of fish	Fish is good for your body	.93
	Fish can substitute meat	
	Fish can help prevent diseases	
	Fish can be used in a variety of dishes	
	Fish is nutritious	
	Fish can be bought in supermarkets	
	Fish is easily digested	
Cons of fruit	Eating fruits is unhandy	.81
	It is hard to find tasty fruits	
	It is difficult to store fruits	
	Eating fruits is tasteless	
	Eating fruits is expensive	
	Fruit spoils quickly	
	Recommendations for fruit are unclear	
	It takes time to buy fruits	
	Chemicals on fruit worry me	
Cons of vegetable	Eating vegetables is unhandy	.82
	It is hard to find tasty vegetables	
	It is difficult to store vegetables	
	Eating vegetables is tasteless	
	Eating vegetables is expensive	
	Vegetables spoil quickly	
	Recommendations for vegetables are unclear	
	It takes time to prepare vegetables	
	It is difficult to prepare vegetables	
	Chemicals on vegetables worry me	
Cons of fish	Eating fish is tasteless	.84
	Fish has an unpleasant smell	
	It is difficult to store fish	
	The bones in fish are unpleasant	
	It is difficult to find good quality fish	
	Recommendations for fish are unclear	
	Fish spoils quickly	
	Fish is difficult to prepare	
	Chemicals in fish worry me	
	People I eat with, do not like fish	
	Eating fish is expensive	
**Do you think it is easy or difficult to eat two servings of fruit each day/200 grams of vegetables each day/fish once or twice a week**		
Self-efficacy for fruit	In the weekends	.90
	During working days	
	In winter	
	When you don't have much time	
	When you experience (emotional) distress	
	When having a touch of flu or a cold	
Self-efficacy for vegetables	In the weekends	.85
	During working days	
	In winter	
	When you don't have much time	
	When you experience (emotional) distress	
	When having a touch of flu or a cold	
Self-efficacy for fish	When you don't have much time	.88
	When there is a lot of choice in fish	
	When the people you eat with do not like fish	
	When you have to prepare the fish yourself	
	When there are still bones in the fish	
	When you do not eat at home, for example at a restaurant or by friends	
